# Single dose of 5 Gy can damage erythrocytes and consequently induces lymphocyte depletion in spleen and circulating blood

**DOI:** 10.3389/fimmu.2026.1848576

**Published:** 2026-06-19

**Authors:** Peimeng You, Xiaohang Qin, Yinghui Li, Huiling Ye, Yuhui Zhao, Guangshan Wang, Cuimeng Tian, Shuo Yin, Junyu Li, Teng Ma, Feng-Ming (Spring) Kong, Jian-Yue Jin

**Affiliations:** 1 School of Biomedical Engineering, Capital Medical University, Beijing, China; 2 Department of Radiation Oncology, Beijing Chest Hospital, Capital Medical University, Beijing, China; 3 Department of Radiation Oncology, Jiangxi Key Laboratory of oncology, Jiangxi Cancer Hospital (The Second Affiliated Hospital of Nanchang Medical College), Nanchang, Jiangxi, China; 4 Cancer Research Center, Beijing Chest Hospital, Beijing Tuberculosis and Thoracic Tumor Research Institute, Capital Medical University, Beijing, China; 5 Department of Clinical Oncology, Hong Kong University Shenzhen Hospital, Shenzhen, China; 6 Department of Clinical Oncology, Queen Mary Hospital, Li Ka Shing Medical School, The University of Hong Kong, Hong Kong, Hong Kong SAR, China

**Keywords:** erythrocyte damage, hemosiderin-laden macrophages, immune microenvironment, radiation-induced lymphopenia, splenic clearance

## Abstract

**Purpose:**

Radiation-induced lymphopenia (RIL) has been well known to associate with poor patient prognosis. Based on interesting findings in our previous study, we hypothesize that a low dose of 5-Gy can damage erythrocytes, which indirectly depletes lymphocytes in spleen, and may contribute to RIL. This study aimed to verify the hypothesis.

**Methods:**

Rats were divided into non-irradiated and 5Gy-irradiated groups. Peripheral blood was collected. Erythrocytes were isolated, irradiated for the irradiated group, and transfused back into the same rat for both groups. Peripheral blood samples were collected at different timepoints post-transfusion for analysis. At 72-hour timepoint, peripheral blood and spleen tissues were collected for flow cytometric analysis and histopathological examination. Morphological changes of irradiated erythrocytes were also studied.

**Results:**

Irradiation of 5–6 Gy could damage erythrocytes in a degree, as demonstrated by morphological changes after radiation. Irradiated and transfused erythrocytes were recognized and phagocytosed in spleen, as demonstrated by significantly increasing hemosiderin-laden macrophages presented in the red pulp. Cytometric analysis and complete blood count analysis showed that the clearing process depleted B lymphocytes in peripheral blood, and both T and B lymphocytes in spleen.

**Conclusions:**

This exploratory study suggests that low-dose irradiated erythrocytes can be recognized and cleared in spleen, and that this process is associated with marked lymphocyte depletion in both the spleen and peripheral blood.

## Introduction

Radiation-induced lymphopenia (RIL) represents one of the most common and severe hematologic adverse effects following tumor radiotherapy and has been well established as a factor closely associated with poor patient prognosis (1). Extensive clinical studies have demonstrated that RIL is significantly correlated with shortened overall survival, increased risk of disease progression, and elevated incidence of distant metastasis in various solid tumors including lung cancer, esophageal cancer, and pancreatic cancer (2, 3). As the core effector cells of anti-tumor immune responses, the sharp decline in lymphocyte numbers not only compromises immune surveillance against tumor cells but may also diminish the efficacy of novel immunotherapeutic agents such as immune checkpoint inhibitors (4, 5).

Currently, the development of RIL is believed to be primarily related to direct radiation-induced killing of circulating lymphocytes during radiotherapy. Lymphocytes are among the most radiosensitive cell types in the body, with a D50 of approximately 2 Gy (6). During conventional fractionated radiotherapy, organs rich in circulating blood such as the heart, lungs, and major vessels are continuously irradiated, resulting in large numbers of circulating lymphocytes receiving cumulative radiation doses and undergoing apoptosis or necrosis (7, 8). In recent years, dosimetric studies have further confirmed that parameters including lung V5, heart V5, mean lung dose, mean heart dose, and estimated dose to immune cells (EDIC) are closely associated with RIL risk (9, 10), suggesting that radiation exposure of the circulating blood pool is a major driver of RIL (11). However, the pathological mechanisms of RIL may be far more complex than simple direct lymphocyte killing, involving multiple interacting factors including bone marrow hematopoietic suppression, lymphoid organ damage, and systemic immune microenvironmental disturbances.

Interestingly, our recent study showed that 5–20 Gy of FLASH radiation with ultra-high dose rate to a mice hind limb depleted much more lymphocytes in spleen than the conventional dose rate with the same dose (12). To explain this interesting phenomenon, we hypothesized that radiation damage to erythrocytes might be responsible for the lymphocyte depletion in spleen. This hypothesis is based on the following considerations: 1) The difference between FLASH and conventional radiation is that FLASH delivers almost entire radiation dose to the small blood volume in the limb in less than 0.4 seconds, while conventional radiation delivers a much smaller dose to a much larger blood volume as blood continuously flow through the irradiated limb (12), consequently, FLASH damages much more erythrocytes as they are quite radioresistant. 2) Radiation-damaged erythrocytes may be recognized and cleared out by macrophages in spleen (13), and the phagocytosis process may trigger inflammatory cytokine release and thus may consume lymphocytes. Given the enormous quantity of erythrocytes in comparison to lymphocytes, the scale of their radiation-induced damage and its indirect effects on the immune system cannot be ignored (14). The purpose of this study was to test the above hypothesis that radiation damage to erythrocytes is the cause of the lymphocyte depletion in spleen. Specifically, we aimed to test whether a radiation dose as low as 5 Gy can generate erythrocyte damage, subsequently induce splenic clearance of the damaged erythrocytes, and finally in the absence of direct lymphocyte irradiation, trigger lymphocyte depletion via splenic clearance. To this end, erythrocytes were separated from blood samples drawn from rats, irradiated, and transfused back into the same rats. Lymphocyte subsets in peripheral blood and spleen were then monitored and compared with those in non−irradiated controls.

## Materials and methods

### Animals

Male Sprague-Dawley (SD) rats (8–10 weeks old, weighing 220–280 g) were purchased from Beijing Vital River Laboratory Animal Technology Co. Ltd. and housed under specific pathogen-free conditions with a 12-hour light/dark cycle at 22 ± 2 °C and 50-60% humidity. Rats had free access to standard chow and water. All animal experiments were approved by the Animal Welfare Committee of Capital Medical University (Approval No. AEEI-2025-253) and conducted in accordance with the Regulations on Animal Experimentation and Laboratory Animal Management of Capital Medical University.

### Experimental design

**Figure 1:**
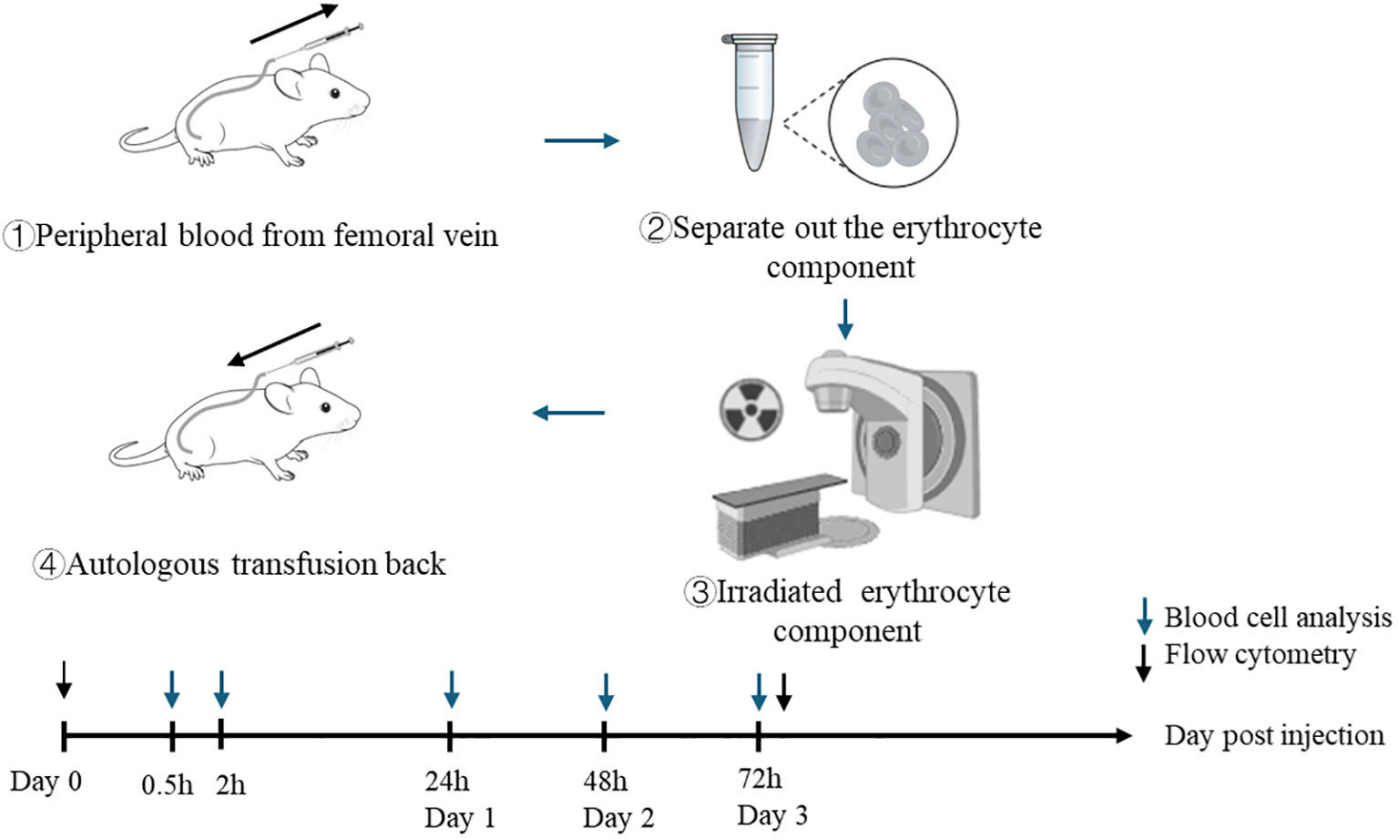
Experimental setup and timepoints for immune response analysis. The experimental process is divided into four steps: ①Collection of peripheral blood from the femoral vein.②Separation of erythrocytes components from whole blood. ③Irradiation of erythrocytes components. ④Autologous transfusion back into the rat. Erythrocytes (irradiated or non-irradiated) are intravenously injected (iv) into the recipient rat. Blood samples are collected at 0, 0.5, 2, 24, 48, and 72 hours after transfusion for hematology analysis. Blood and spleen samples are collected at 72 hours for flow cytometry analysis.

This study employed an autologous transfusion model to avoid immune rejection. The experimental workflow is shown in **Figure 1**: Rats were randomly divided into two groups: a 5-Gy irradiated group (IR_5Gy) and a non−irradiated control group (Non_IR). The Non_IR group underwent the same procedures as the IR_5Gy group, including femoral vein catheterization, blood collection (1 mL), centrifugation, erythrocyte separation, three washes with sterile PBS, and autologous transfusion back into the same rat. The only difference was that the isolated erythrocytes were not exposed to radiation (sham irradiation, placed in the same room for the same duration as the irradiated group). The processed erythrocytes were transfused back into the same rat through the femoral vein catheter in approximately 2 hours. Small blood samples were collected through the catheter before transfusion (0 h) and at five time points post-transfusion (0.5 h, 2 h, 24 h, 48 h, 72 h) for routine blood testing. At the experimental endpoint (72 h), rats were euthanized, and peripheral blood and spleen tissues were collected for flow cytometric analysis to assess the immunological change after transfusion of the erythrocytes. In addition, some erythrocyte samples from same rat were irradiated with 5, 6, 12 and 24 Gy to study radiation induced morphological changes.

### Irradiation

Erythrocytes collected from the rats were irradiated using a Varian X-ray linear accelerator at Beijing Chest Hospital Radiation Oncology Department at a dose rate of 1 Gy/min, with a field size of 10 cm x10 cm.

### Femoral vein catheterization

Rats were anesthetized by intraperitoneal injection of 2.5% tribromoethanol (1 mL/kg). The right femoral vein was surgically exposed and catheterized with a 0.20 mm polyurethane catheter. The catheter was tunneled subcutaneously to the dorsal cervical region, secured with a vascular access button, and flushed daily with heparinized saline (100 IU/mL) to maintain patency.

### Blood collection and processing

Peripheral blood (1 mL) was collected through the femoral vein catheter into EDTA anticoagulant tubes. Erythrocytes were separated by centrifugation at 1200 rpm for 10 minutes at 4 °C, then washed three times with sterile phosphate-buffered saline (PBS). Following each washing step, the buffy coat was meticulously removed to minimize the contamination by leukocytes.

### Autologous erythrocyte transfusion

Erythrocytes meeting quality criteria were transfused autologously through the femoral vein catheter. Based on an estimated blood volume of 16 mL for a 250 g rat, the transfused volume represented approximately 6% of the total circulating blood volume. Therefore, the expected proportion of erythrocytes in peripheral circulation was approximately 6%. The above procedures include separating erythrocytes from whole blood, washing, irradiation, and autologous blood transfusion, all of which are performed rapidly under sterile conditions.

### Blood collection and complete blood count analysis

At 0.5, 2, 24, 48, and 72 hours post-transfusion, peripheral blood samples (~50 μL) were collected through the femoral vein catheter. In addition, a small amount of blood (~50 μL) was also collected at the time for collecting the transfusion blood for each rat and serves as the control for non-transfusion and the baseline at time 0. Complete blood counts including white blood cells (WBC), lymphocytes (LYM), monocytes (Mon), granulocytes (Gran), and red blood cells (RBC) were determined using an automated hematology analyzer (Countstar, Mira BF).

### Tissue collection and processing

At 72 hours post transfusion, rats were euthanized by cervical dislocation under isoflurane anesthesia. The spleen was dissected, weighed, and processed for subsequent analysis. The spleen was minced and passed through a 70 μm cell strainer (BD Biosciences, Franklin Lakes, NJ, USA) to generate a single-cell suspension. Erythrocytes were lysed using ACK lysis buffer (Thermo Fisher Scientific) for 5 minutes at room temperature. Cells were washed three times with PBS and resuspended in PBS at a concentration of 1×10^7 cells/mL.

### Flow cytometry immunophenotyping

Single-cell suspensions were stained with fluorochrome-conjugated antibodies against the following surface markers: CD3 (T cells), CD4 (helper T cells), CD8 (cytotoxic T cells), CD45 (leukocytes), CD45RA (B cells), and CD68 (macrophages). All antibodies were purchased from BioLegend (USA). Supplementary Material for specific details. Samples were incubated with antibodies for 30 minutes at 4 °C in the dark, washed twice with FACS buffer, and resuspended in PBS for analysis. Data were acquired on a BD FACSCanto™ II flow cytometer. At least 10,000 CD45^+^ events were acquired per sample. Lymphocytes were first gated by FSC-A vs. SSC-A, followed by FSC-A vs. FSC-H doublet discrimination, then CD45^+^ leukocytes, and finally by lineage markers (CD3, CD4, CD8, CD45RA, CD68). Representative gating plots are shown in **Supplementary Figure 3**. Data were analyzed using FlowJo software. Absolute cell counts were calculated based on flow cytometry percentages and total cell counts determined by hematology analyzer.

### Histological analysis

Spleen tissues were fixed in 4% paraformaldehyde for 24 hours, embedded in paraffin, and sectioned at 5 μm thickness. Sections were deparaffinized, rehydrated according to standard protocols, and stained with hematoxylin and eosin (H&E). Images were acquired using a Leica DM3 XL microscope at 200× or 400× magnification.

### Erythrocyte morphological analysis

Fresh blood smears were prepared on glass slides, air-dried, and stained with Wright-Giemsa stain. Erythrocyte morphology was examined under a Leica DM3 XL microscope. Abnormal erythrocyte morphologies were identified and quantified by counting at least 200 erythrocytes per sample. Additionally, erythrocytes were fixed in PBS containing 2.5% glutaraldehyde at 4 °C for 0.5 hours, dehydrated through a graded ethanol series, and subjected to critical point drying. Samples were sputter-coated with gold-palladium and observed using a scanning electron microscope S-4800 at an accelerating voltage of 10 kV.

### Statistical analysis

Each experimental group consists of n=3 rats, covering all aspects of whole blood cell count, flow cytometry, and histological analysis. For quantitative analysis of red blood cell morphology, we analyzed at least 200 red blood cells per sample from three independent experiments. Data are presented as mean ± standard deviation or median with interquartile range, as appropriate. Statistical comparisons between two groups were performed using unpaired Student’s t-test or Mann-Whitney U test for non-parametric data. Multiple group comparisons were analyzed by one-way ANOVA followed by Tukey’s *post-hoc* test. The comparison across multiple time points was conducted using bidirectional repeated ANOVA, followed by multiple comparison test. Paired data were analyzed using paired t-test. Cohen’s d analysis was used to test the effective sizes. Statistical significance was defined as *P < 0.05, **P < 0.01, and ***P < 0.001. All analyses were performed using R version 4.4.0.

## Results

### Radiation induces morphological changes in erythrocytes

**Figure 2:**
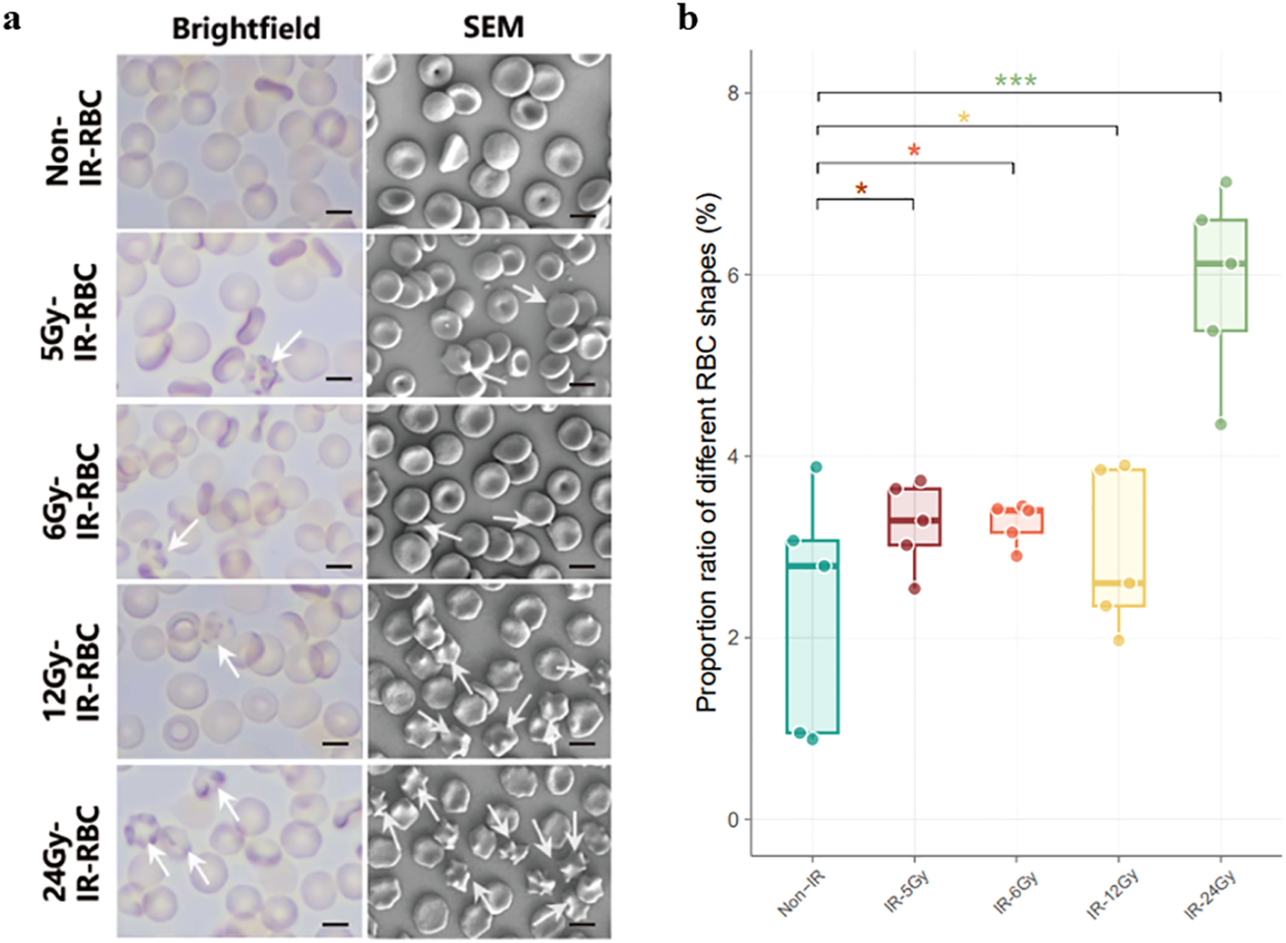
Morphological changes of RBCs after irradiation. **(a)** Representative brightfield microscopy and scanning electron microscopy images of RBCs: Non-irradiated RBCs exhibited typical biconcave disc morphology; after irradiation with 5 Gy, 6 Gy, 12 Gy, and 24 Gy, RBCs showed progressive morphological alterations including echinocyte formation (arrows), membrane blebbing, and spherocyte transformation (scale bars represent 10 μm). **(b)** The proportion of RBCs with abnormal shapes was significantly increased in irradiated groups compared to non-irradiated controls. Data are presented as box plots with individual data points. *p < 0.05, ***p < 0.001 compared to Non-IR group.

Peripheral blood erythrocytes from SD rats irradiated with 5 Gy, 6 Gy, 12 Gy, and 24 Gy were examined by brightfield microscopy and scanning electron microscopy (SEM). As shown in **Figure 2**, non-irradiated (Non_IR) erythrocytes displayed typical biconcave discoid shapes with smooth cell margins and clear central pallor under brightfield microscopy. SEM observation revealed smooth cell surfaces with uniform morphology and regular biconcave discoid structures. In contrast, irradiated groups showed progressively worsening erythrocyte morphological abnormalities with increasing radiation dose. Under brightfield microscopy, numerous erythrocytes exhibited increased cell volume and irregular morphology. SEM images demonstrated a significant increase in the proportion of abnormally shaped erythrocytes, with obvious echinocyte formation and aggregation of dysmorphic cells. Statistical analysis of erythrocyte abnormality rates revealed a clear dose-effect relationship of X-ray-induced peripheral blood erythrocyte damage. Low-dose (5–6 Gy) irradiation caused mild elevation of erythrocyte abnormality rates with statistical significance (P < 0.05), while at 24 Gy, erythrocyte abnormality rates increased significantly to more than 2-fold higher than the non-irradiated group (P < 0.001). However, no cell hemolysis was observed under these irradiation conditions (**Supplementary Figure 1**).

### Femoral vein catheterization and erythrocyte transfusion do not affect rat physiological status

**Figure 3:**
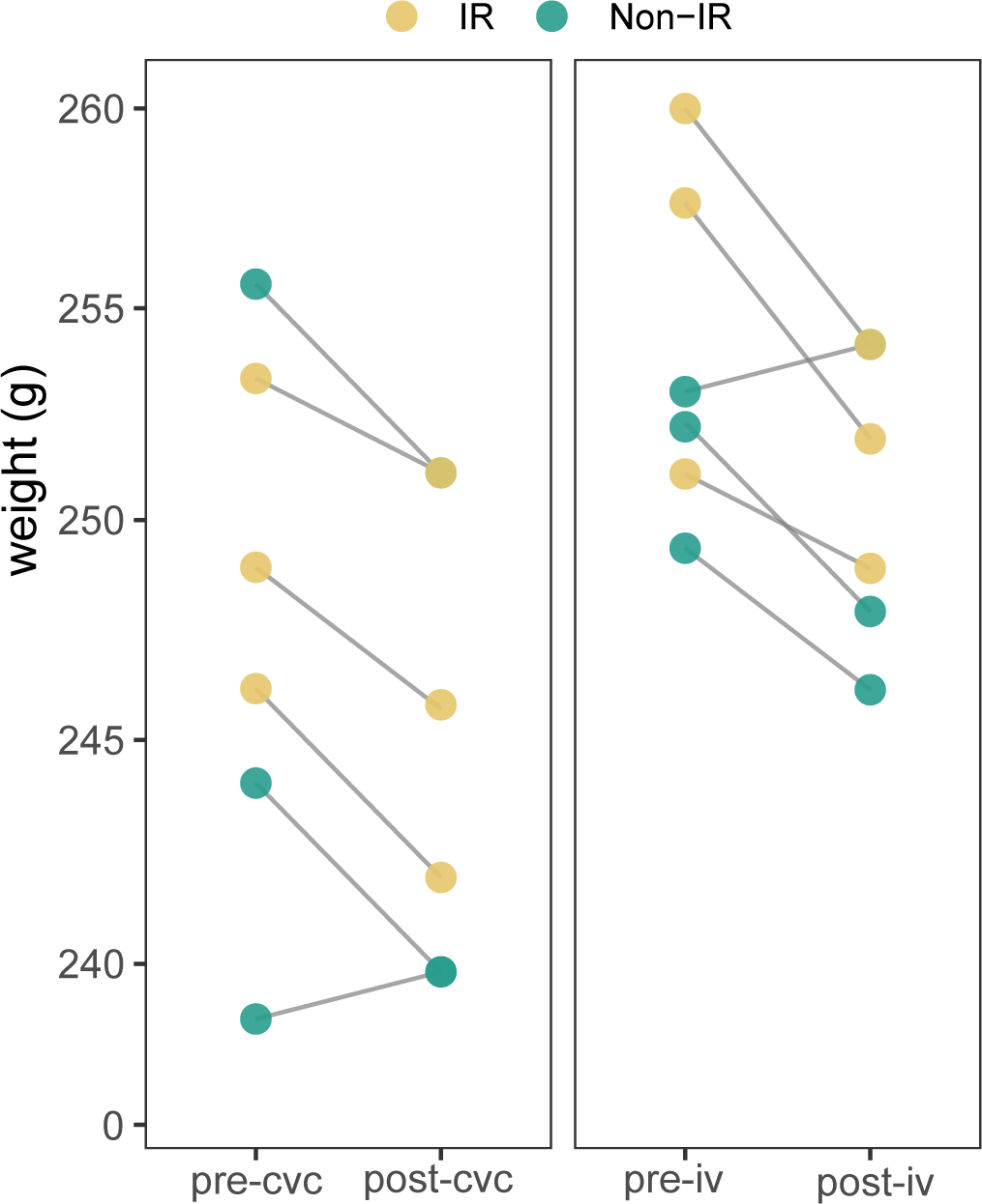
Body weight changes after RBCs transfusion. Comparison of body weight changes in rat receiving Non-IR or IR RBCs via femoral vein catheter or intravenous injection. Data are presented as individual data points with box plots. Defined “cvc” as catheterization “after catheter placement but before transfusion” and “iv” as “after intravenous transfusion”.

To evaluate the impact of femoral vein catheterization and erythrocyte transfusion procedures on rat physiological status, we monitored changes in body weight before and after the experiment. As shown in **Figure 3**, rat body weight showed no significant changes after either catheterization or transfusion procedures compared to pre-procedure measurements. Paired data points showed stable body weight before and after procedures, with no significant differences between irradiated and non-irradiated groups. These results indicate that femoral vein catheterization and erythrocyte transfusion procedures have no significant effects on rat body weight. The experimental model demonstrates good stability and reproducibility, excluding interference from surgical trauma and procedural stress on subsequent erythrocyte clearance kinetic analyses.

### Irradiated erythrocytes are cleared by macrophages in spleen

**Figure 4:**
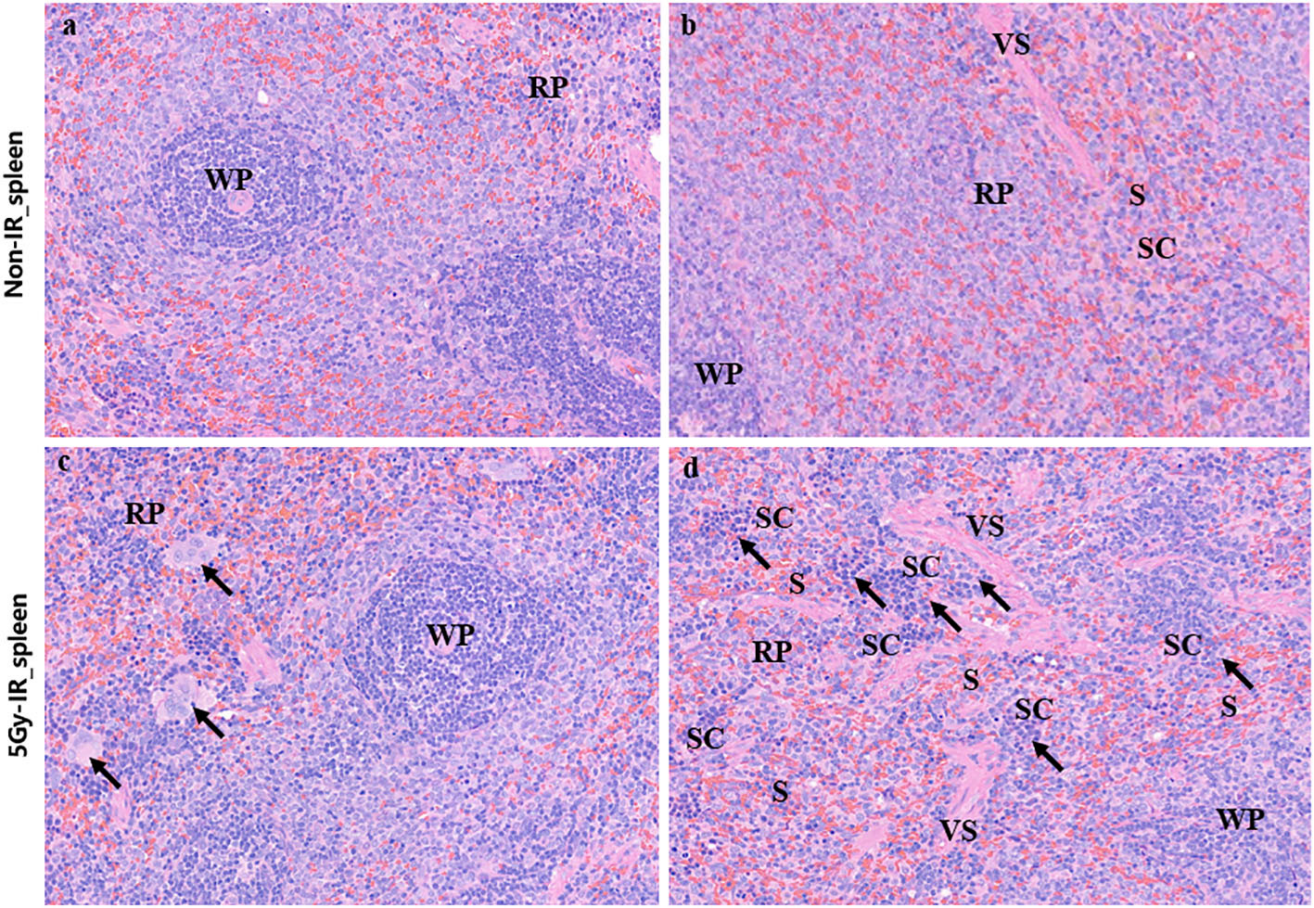
Demonstration of radiation-damaged red blood cells (RBCs) being recognized and phagocytosed in spleen. Histopathological changes in spleen after transfusion of irradiated RBCs are noted by comparing H&E staining of spleen 72h post-transfusion between 5Gy-irradiated and non-irradiated groups. **(a)** White pulp and its neighborhood for Non-IR group: clear demarcation between the white pulp and the red pulps with well-defined lymphoid follicles around the white pulp, no evidence of hemosiderin-laden macrophages in the entire spleen. **(b)** Red pulps for Non-IR group: normal splenic cords, splenic sinusoids and vascular structure with moderate cell density in the red pulp. **(c)** White pulp and its neighborhood for IR_5Gy group: multiple hemosiderin-laden macrophages are presented in the neighborhood of white pulps (marked by arrows) for the irradiated group, indicating that damaged-RBCs were engulfed by the macrophages. **(d)** Red pulps for IR_5Gy group: increased congestion of splenic cords, splenic sinusoids and vascular structures in the red pulps (marked by arrows), possibly due to blockage by a large number of damaged RBCs. Scale bars represent 50 μm. RP, red pulp; WP, white pulp; SC, splenic cords; S, Splenic sinusoid. VS, Vascular structure.

We performed histopathological examination of spleen tissues 72 hours post transfusion using H&E staining. The spleen displayed normal tissue architecture with clear demarcation between white pulp and red pulp for the Non_IR group, and well-defined lymphoid follicles with densely packed lymphocytes were visible within the white pulp (**Figure 4a**). The red pulp showed normal sinusoidal structures with moderate cell density (**Figure 4b**). In contrast, for the IR_5Gy group, the spleen showed mild congestion of sinusoidal/vascular structures and increased cell density in the red pulp (**Figure 4d**), with destruction of lymphoid follicle architecture and reduced lymphocyte density in the white pulp (**Figure 4c**). Interestingly, many multinucleated giant macrophages appeared in the red pulp near the white pulp (**Figure 4c**). These giant cells are uniquely characterized by their golden−brown granular cytoplasmic pigment and multinucleated morphology, consistent with hemosiderin-laden macrophages as the result of phagocytosis of radiation-damaged erythrocytes. We have quantified these cells in 5 random high−power fields (400×) for each animal. An average of 10 cells were observed in each field in the IR_5Gy group. In contrast, only in the Non_IR group (P = 0.019, **Supplementary Table 1**). The presence of these cells suggests that 5 Gy irradiation can generate significant damage to erythrocytes, and these damaged erythrocytes can be cleared out by phagocytosis in the spleen.

### Irradiated erythrocytes are associated with time-dependent changes in other blood cells

**Figure 5:**
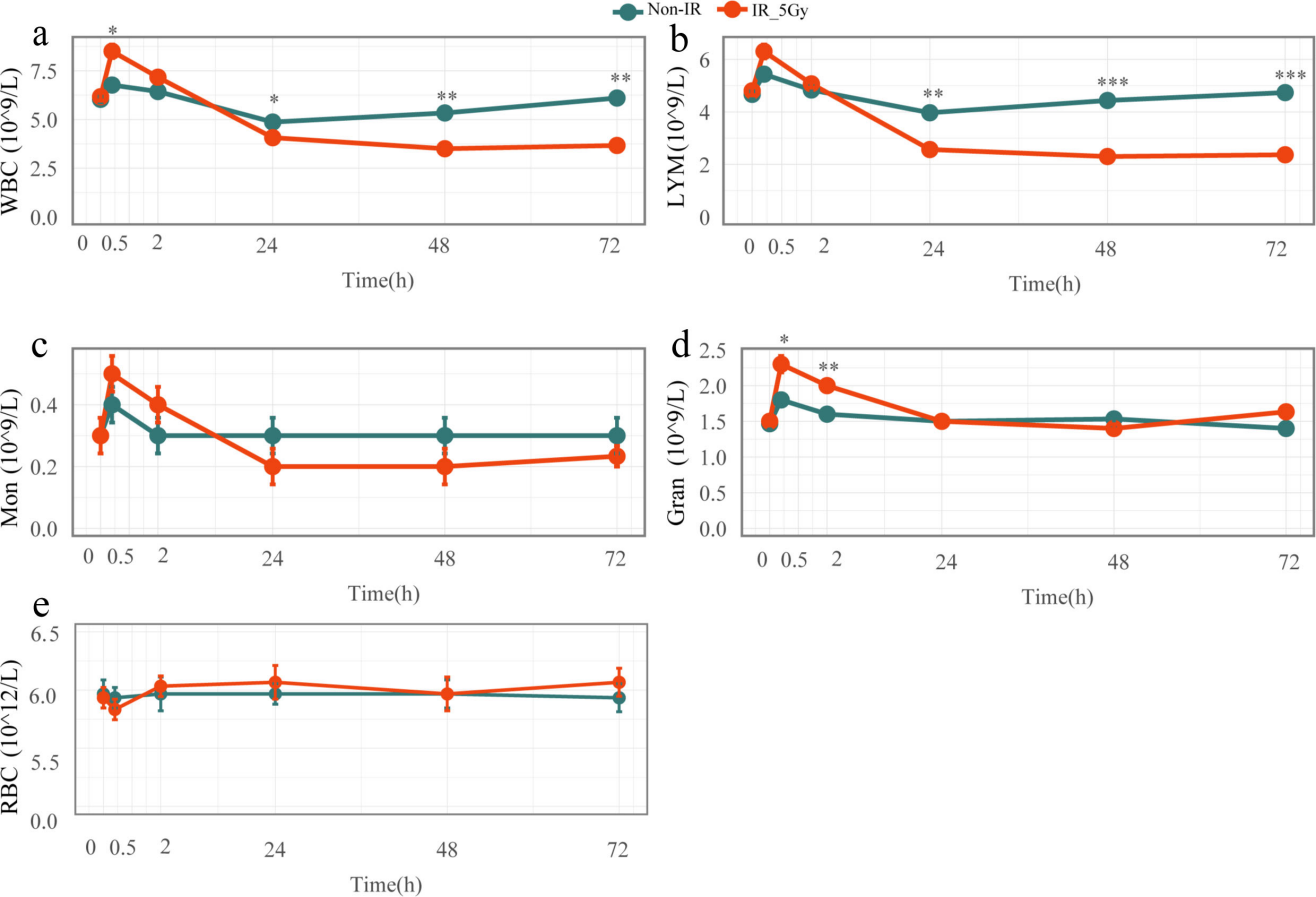
Irradiated red blood cells (RBCs) induces reduction of white blood cells, lymphocytes and monocytes in peripheral blood. These reductions are observed by comparing hematological parameters, including blood counts of WBC **(a)**, lymphocytes **(b)**, monocytes **(c)**, granulocytes **(d)**, and red blood cells **(e)** in peripheral blood between IR_5Gy and Non-IR groups 0.5, 2, 24, 48 and 72 hours following transfusion. The counts of WBCs (P < 0.05), lymphocytes (P < 0.001) for the IR_5Gy group are significantly lower than that in the Non-IR group after 24 h. A transient increase of WBCs, lymphocytes, monocytes and granulocytes is observed at 0.5 hours after transfusion for the IR_5Gy group. Data are presented as mean ± SEM. Between-group comparisons at each time point are indicated in the figure: *P < 0.05, **P < 0.01, ***P < 0.001.

**Figure 5** shows the variations of various blood cells at different time points after transfusion of irradiated and non-irradiated erythrocytes back to the hematopoietic system. Statistical comparisons between the irradiated and non−irradiated groups at each time point, and between different time points in each group, are presented in **Supplementary Table 2**. For the IR_5Gy group, the WBCs, lymphocytes, monocytes and granulocytes all showed a transient elevation at 0.5 hours post-transfusion, suggesting that irradiated erythrocytes induced an inflammation response. These cells quickly decreased to a significantly lower level at 24 hours in comparison to the baseline and kept at a similarly low level at 48–72 hours, except for the granulocytes, suggesting that 5 Gy of radiation induced significant damage in the erythrocytes, and consequently caused depletion of WBC, lymphocytes and monocytes in peripheral blood. The granulocytes decreased to the baseline level at 24 hours and kept at the similar level at 48–72 hours.

On the other hand, all these cells for the non-irradiated group exhibited only a mild, transient increase at 0.5 hours, decreased to a level slightly lower than the baseline (WBC and Lymphocyte) or similar to the baseline (monocyte and granulocyte) at 24 hours, and finally recovered to the baseline level at 72 hours, suggesting that the erythrocyte washing and transfusion process may also induce a mild effect on the erythrocytes similar as the radiation. There were significant differences between IR_5Gy and Non_IR groups at each time point for the WBC, lymphocytes and monocytes, indicating that just 5 Gy of radiation to the erythrocytes can induce significant reduction of these cells in peripheral blood. The erythrocyte level was relatively stable at different time points for both groups. It dipped slightly (no significant) at 0.5 hours, corresponding to the transient elevation of other blood cells.

### Irradiated erythrocytes associated with lymphocyte depletion in spleen and peripheral blood

**Figure 6:**
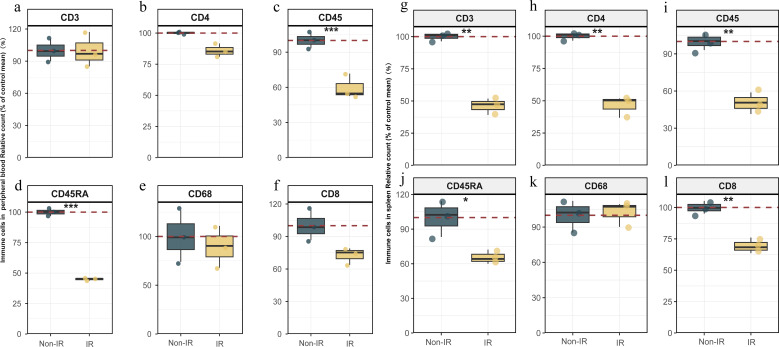
Irradiated red blood cells induce relative reduction of B lymphocytes in peripheral blood, and T and B lymphocytes in spleen. Comparisons of cell counts for CD3^+^ T cells **(a)**, CD45RA^+^ B cells **(b)**, CD4^+^ helper T cells **(c)**, CD68^+^ macrophages **(d)**, CD45^+^ leukocytes **(e)**, and CD8^+^ cytotoxic T cells **(f)** in peripheral blood, and corresponding cells **(g-l)** in spleen 72 hours post transfusion. To highlight relative changes, data are normalized to the mean of the Non_IR group (set as 100%). Boxes represent interquartile range, whiskers show min to max, and dots indicate individual animals. Statistical comparisons between groups were performed on absolute counts using unpaired t-test. *P<0.05, **P < 0.01, ***P < 0.001. Raw absolute counts for each animal are shown in **Supplementary Figure 4**.

**Figure 6** shows the comparison of the normalized flow-cytometry-measured immune cell subset population 72 hours post-transfusion between the Non_IR group and the IR_5Gy group in peripheral blood and spleen. The WBC (CD45+), T lymphocytes (CD3+), CD4+ helper T cells, CD8+ cytotoxic T cells, and B lymphocytes (CD45RA+) all had significantly lower counts for the IR_5Gy group in the spleen. On the other hand, only the WBC (CD45+) and B lymphocytes (CD45RA+) had significant lower counts for the IR_5Gy group in the peripheral blood. Interestingly, the B lymphocytes had more severe reduction in the peripheral blood (P<0.001, reduced to 45%) than in the spleen (P<0.05, reduced to 62.5%) after erythrocyte irradiation, while T lymphocytes had more severe reduction in the spleen (P<0.01, reduced to 49%) than in the peripheral blood (not significant, reduced to 97%). The macrophage counts (CD68+) appeared to have no changes between the two groups in both spleen and peripheral blood. Comparisons of absolute cell counts for all subsets between the two groups showed same results as normalized cell counts (**Supplementary Figure 4**). A Cohen’s effective size and 95% confidence intervals analysis indicated that the effective sizes are much larger than 0.8 for CD45, CD4, CD8, CD45RA in peripheral blood, and for CD45, CD3, CD4, CD8, CD45RA in spleen (**Supplementary Table 3**), indicating that our results are solid even with a relatively small sample size.

## Discussion

This study demonstrates our hypothesis that erythrocyte irradiation with a dose as low as 5 Gy is associated with lymphocyte depletion in the spleen and peripheral blood, suggesting that low-dose radiation can damage erythrocytes in a certain degree, these damaged erythrocytes can be recognized and cleared in the spleen, and the clearance process may be associated with lymphocyte depletion. This statement is based on the following findings: 1) An *in vitro* study showed that radiation induced significant dose-dependent morphological changes in erythrocytes; even at a dose as low as 5 Gy, the morphological changes were detectable; 2) Transfusion of 5Gy-irradiated erythrocytes back to the circulating blood resulted in increasing hemosiderin-laden macrophages in spleen 72 hours post transfusion, suggesting that significant amounts of irradiated erythrocytes were damaged, and they were recognized and phagocytosed in the spleen; 3) Transfusion of these erythrocytes was associated with significant depletion of lymphocytes in peripheral blood and spleen. These findings are significant because they support an important hypothesis regarding erythrocyte-mediated RIL and may open a valuable new direction in radiation immunobiology.

RIL is well established as a factor relating to radiotherapy outcome. Direct radiation damages to the lymphocytes in circulating blood and other lymphoid organs has been considered as the major cause of RIL. Although radiation induced anemia has been reported (15), the damaging effects of ionizing radiation on erythrocytes have never been considered to be related to RIL. In this study, we have observed that single fraction of just 5 Gy to only ~6% of erythrocytes caused lymphocyte depletion to less than 50% of the baseline level in peripheral blood, and the depletion could last for quite long time. Generally, conventional radiotherapy delivers 60–70 Gy fractionated dose to an irradiated volume 5-20% of the total body volume. Assuming an irradiated volume of 10% of the body volume with an average dose of 60 Gy, it will give the entire circulating blood an average dose approximately to 6 Gy, with a part of blood volume receiving much higher doses. Because the erythrocytes do not have a nucleus, their repair capability is poor. Therefore 6 Gy accumulated dose from multiple fractions would generate a similar amount of damages as 6 Gy in single fraction. Thus, it is reasonable that radiation damages to the erythrocytes in a radiotherapy course can cause much more severe lymphocyte depletion than what have been observed in this study, and this erythrocyte-associated lymphocyte depletion may be one of the important factors contributing to clinical RIL, but this hypothesis requires testing in fractionated irradiation models and clinical correlative studies.

Ionizing radiation can induce membrane lipid peroxidation, membrane protein oxidative damage, and cytoskeletal protein cross-linking, leading to decreased erythrocyte membrane fluidity and deformability (13, 16, 17). Radiation-induced reactive oxygen species can attack unsaturated fatty acids in membrane phospholipids, triggering lipid peroxidation chain reactions that lead to increased membrane permeability and ion balance disturbances (18). These radiation damages are similar to the erythrocyte aging process, except that the irradiation greatly accelerates the process. It is well documented that aged erythrocytes are detected and cleared in spleen and liver (19, 20). Normal erythrocytes have a lifespan of approximately 120 days. Thus, a 10 seconds blood circulation cycle will clear approximately 0.0001% of erythrocytes. Assuming that 5 Gy irradiation generating 1% aging/damaging erythrocytes, it will require the immune system to clear 10000 times more erythrocytes than a normal condition. Therefore, it is understandable that we observed significantly increasing hemosiderin-laden macrophages and sinusoid congestion in spleen after transfusion of irradiated erythrocytes. Interestingly, we also observed mainly T lymphocyte depletion in spleen, and B lymphocyte depletion in peripheral blood, suggesting different involvements of the T and B cells in clearing out the damaged erythrocytes.

We have also found that there is a transient increase of various blood cells at 0.5 hours post transfusion of irradiated erythrocytes. This increase ranges from ~40% for the WBC and lymphocytes, and up to 67% for the monocytes. Logically, this transient increase should be due to the detection of large number of damaged erythrocytes and consequently releasing a signal to regenerate the required immune cells involved in clearing out the damaged erythrocytes. The monocytes, which are the precursor of macrophages, have the largest increase because macrophages are directly involved in the clearing process. These cells then decrease rapidly at 2 hours and drop to a level significantly lower than the baseline at 24 hours for WBCs, lymphocytes and monocytes, suggesting these cells have been consumed or involved in the clearing process. They keep at a similarly low level at 48 and 72 hours, suggesting that the consumption of these cells reaches to a stable balance. The granulocytes drop to the baseline level at 24 hours, and keep in the similar level later, suggesting that granulocytes may not involve in the clearing process.

This study has limitations. Although the study demonstrated that low-dose irradiation to erythrocytes can lead to lymphocyte depletion, it did not investigate the molecular or cellular mechanisms that link to the erythrocyte-mediated lymphocyte depletion phenomenon. While the observation of hemosiderin-laden macrophages suggests that splenic clearance of damaged erythrocytes may be the middle step associated with the phenomenon, no iron staining with Prussian blue or other erythrophagocytosis markers, and macrophage functional assays were performed to fully elucidate this pathway. More importantly, there is a mechanism gap between the splenic clearance and the lymphocyte depletion. It is not clear how and in what condition the splenic clearance induce the lymphocyte depletion. We have speculated that excessive splenic clearance may cause inflammation, which consequently induce the lymphocyte depletion. However, there this no any experimental data to support it. Future works are required to study 1) whether splenic clearance is indeed the middle step for the phenomenon and what is its detailed pathway; 2) the detailed mechanisms link the splenic clearance to the lymphocyte depletion; 3) the irradiated dose and volume effects to the phenomenon.

## Conclusions

This study supports our hypothesis that erythrocyte irradiation with a dose as low as 5 Gy is associated with lymphocyte depletion in the spleen and peripheral blood. The results suggest that low-dose radiation can damage erythrocytes in a certain degree, these damaged erythrocytes can be recognized and cleared in the spleen, and the clearance process may be associated with lymphocyte depletion.

## Data Availability

The original contributions presented in the study are included in the article/supplementary material. Further inquiries can be directed to the corresponding author.
