# Supplementary material for: Single dose of 5 Gy can damage erythrocytes and consequently induces lymphocyte depletion in spleen and circulating blood
Source: Front Immunol. 2026 Jun 19;17:1848576. doi: 10.3389/fimmu.2026.1848576 (PMC13328184; doi:10.3389/fimmu.2026.1848576)
Supplement: Supplementary file 1 [file DataSheet1.docx]

**Detection of red blood cell agglutination reaction by orifice plate method**

Add 2 × 10^7^ irradiated red blood cells to PBS buffer to obtain the total volume.For a mixture of 200 µ L. Afterwards, add the mixed solution to a 96 well "U" - shaped microreactor plate. The normal red blood cell group was used as the control group, and multiple wells were set up in each group. A 96 well "U" - shaped micro reaction plate was placed in the incubator and incubated at 37 ℃ for 30 minutes. Microscopic images were taken and recorded.

our hemolysis assay (Supplementary Fig. 1) confirmed that 5 Gy causes no significant hemolysis, ruling out non‑specific effects from free hemoglobin or heme.


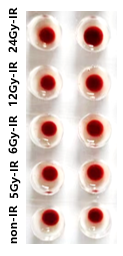


**Supplementary Fig. 1. Results of Peripheral Blood Hemolysis Plate Experiment under Different Dose Groups of Radiation**

Single-cell suspensions were stained with fluorochrome-conjugated antibodies against the following surface markers: CD3 (T cells), CD4 (helper T cells), CD8 (cytotoxic T cells), CD45 (leukocytes), CD45RA (B cells), and CD68 (macrophages). All antibodies were purchased from BioLegend (USA).

**CD3: clone 1F4 (BioLegend, cat# 201417)**

**CD4: clone W3/25 (BioLegend, cat# 201517)**

**CD8: clone OX-8 (BioLegend, cat# 201703)**

**CD45: clone OX-1 (BioLegend, cat# 202218)**

**CD45RA: clone OX-33 (BioLegend, cat# 202305)**

**CD68: clone QA20A71 (BioLegend, cat# 201003)**

Supplementary Table 1. Statistical analysis of differences in white blood cells, lymphocytes, and monocytes in peripheral blood at different time points. Between-group comparisons at each time point are indicated in the figure: *P < 0.05, *p < 0.05, ***p < 0.001.

| **Time** | **WBC(t/P)** | **LYM(t/P)** | **MON(t/P)** | **GRAN(t/P)** | **RBC(t/P)** |
| --- | --- | --- | --- | --- | --- |
| 0 | 0.535/0.624 | 0.632/0.565 | 0.000/1.000 | 0.316/0.770 | -0.224/0.835 |
| 0.5 | 3.803/0.020* | 2.871/0.061 | 1.225/0.288 | 3.873/0.032* | -0.802/0.468 |
| 2 | 2.557/0.063 | 1.136/0.320 | 1.225/0.288 | 4.899/0.008** | 0.392/0.719 |
| 24 | -3.207/0.038* | -8.758/0.002** | -1.225/0.288 | 0.000/1.000 | 0.588/0.594 |
| 48 | -6.875/0.003** | -9.760/0.001*** | -1.265/0.285 | -1.265/0.285 | 0.000/1.000 |
| 72 | -7.529/0.003** | -12.551/<0.001*** | -1.000/0.387 | 2.214/0.102 | 0.784/0.477 |


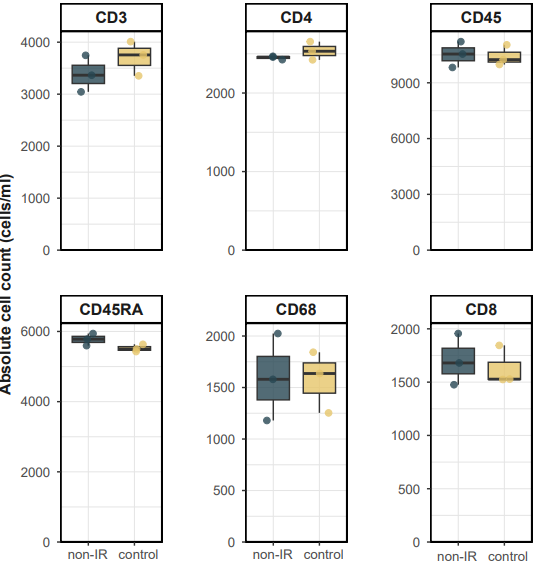

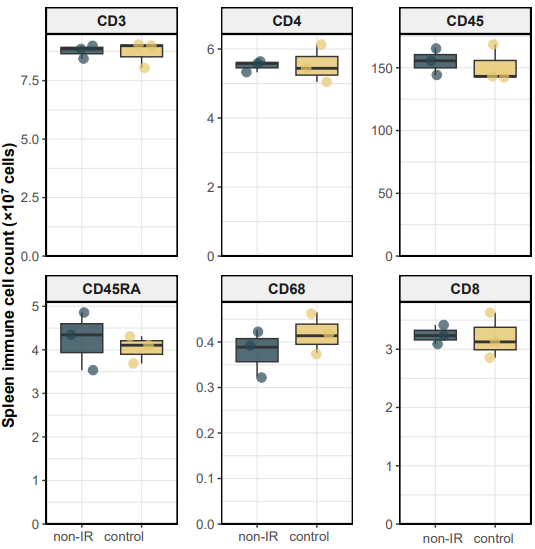


**Supplementary Fig. 2. Absolute immune cell counts of control group (without catheterization or blood transfusion) and non IR group rats. Peripheral blood count is calculated based on the absolute number of immune cells per milliliter, while spleen count is calculated based on a 10^7^ count**

**Supplementary Table 2. Effect sizes (Cohen's d) and 95% confidence intervals for immune cell subsets in peripheral blood and spleen.**

| **Tissue** | **Cell subset** | **Cohen's d** | **95% CI** |
| --- | --- | --- | --- |
| Peripheral blood | CD45 | 4.70 | (0.94, 8.46) |
| Peripheral blood | CD3 | 0.02 | (-2.85, 2.89) |
| Peripheral blood | CD4 | 3.57 | (0.42, 6.72) |
| Peripheral blood | CD8 | 2.41 | (-0.02, 4.84) |
| Peripheral blood | CD45RA | 26.20 | (6.80, 45.6) |
| Peripheral blood | CD68 | 0.41 | (-2.07, 2.89) |
| Spleen | CD45 ×10⁷ | 6.38 | (1.70, 11.1) |
| Spleen | CD3×10⁶ | 10.50 | (3.16, 17.8) |
| Spleen | CD4 ×10⁶ | 9.48 | (2.79, 16.2) |
| Spleen | CD8 ×10⁶ | 5.08 | (1.14, 9.03) |
| Spleen | CD45RA ×10⁶ | 2.80 | (0.18, 5.42) |
| Spleen | CD68 ×10⁶ | -0.18 | (-3.01, 2.65) |


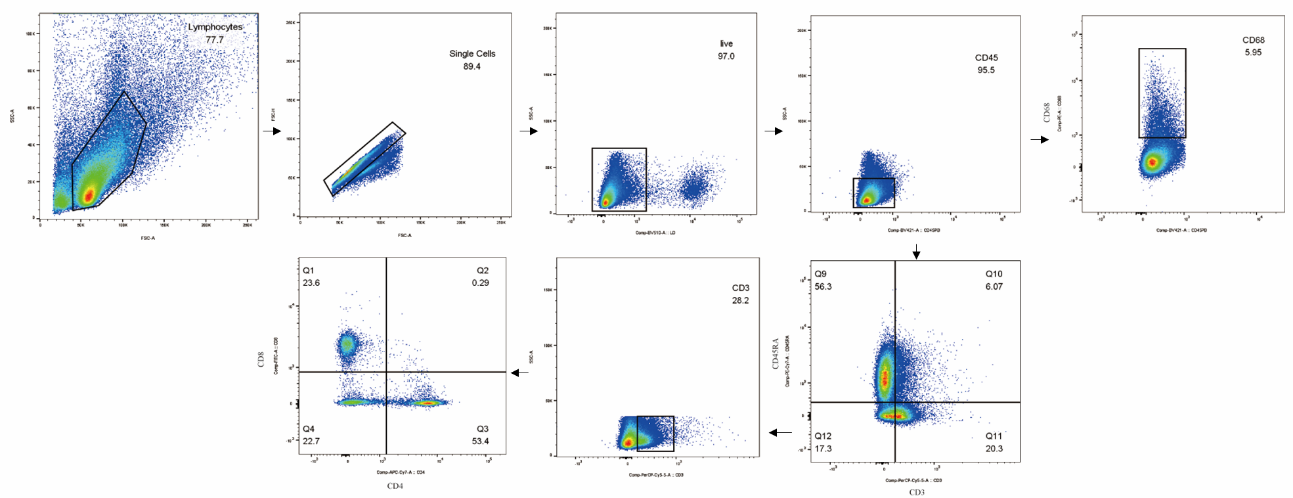


**Supplementary Fig. 3. Representative flow cytometry gating strategy.**


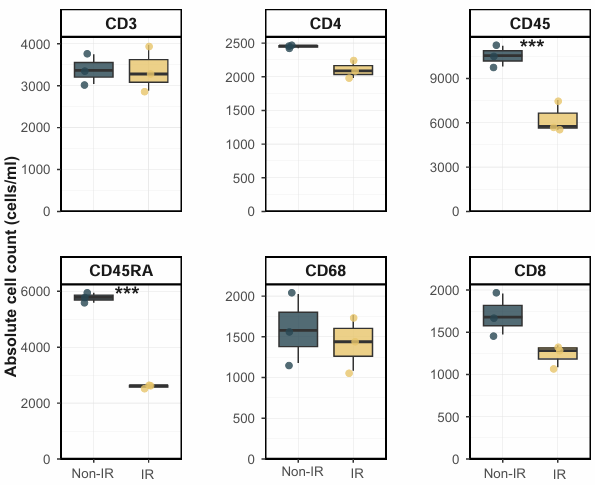


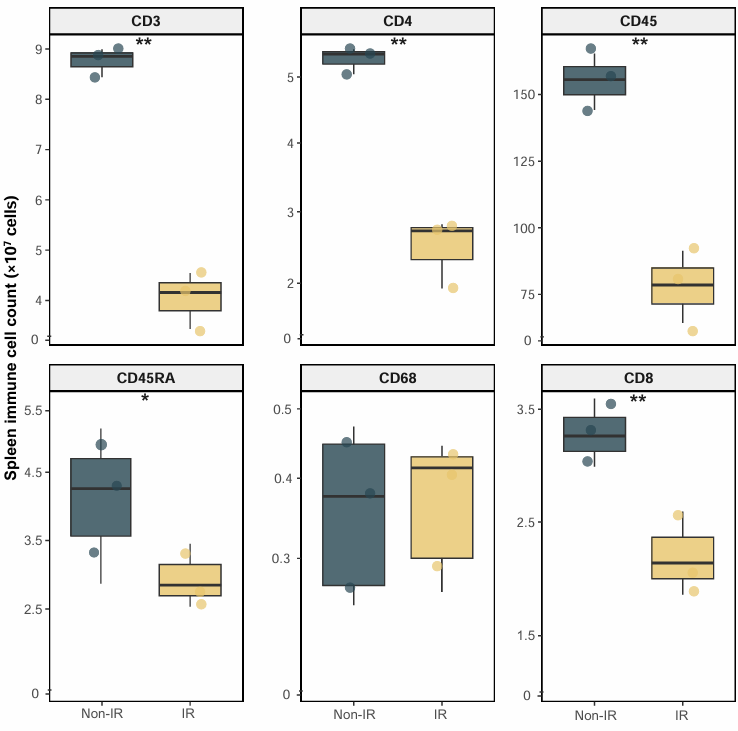


**Supplementary Fig.4.** **Absolute count of peripheral blood and spleen lymphocytes 72 hours after transfusion.**

**Supplementary Table 3. Observation under HE sectioning microscope, counting the number of macrophages containing brown particles in each field of view**

| **Group** | **Cell Count** | ***P*** |
| --- | --- | --- |
| IR_5Gy | 9.8 ± 5.26 | 0.019* |
| Non_IR | 1.0 ± 1.22 |  |
